# Critical view of safety faster and safer technique during laparoscopic cholecystectomy?

**DOI:** 10.12669/pjms.343.14309

**Published:** 2018

**Authors:** Mohammad Zarin, Muhammad Asim Khan, Maryam Alam Khan, Syed Asad Maroof Shah

**Affiliations:** 1 Mohammad Zarin, FCPS. Department of Surgery, MTI, Khyber Teaching Hospital, Peshawar, Pakistan; 2 Muhammad Asim Khan, MBBS. Department of Surgery, MTI, Khyber Teaching Hospital, Peshawar, Pakistan; 3 Maryam Alam Khan, FCPS. Queen Elizabeth Hospital, Birmingham, UK; 4 Syed Asad Maroof Shah, FCPS. Department of Surgery, MTI, Khyber Teaching Hospital, Peshawar, Pakistan

**Keywords:** Bile Duct Injury, Critical View of Safety (CVS), Infundibular technique, Laparoscopic cholecystectomy

## Abstract

**Objective:**

Incidence of Bile Duct Injuries (BDI) during Laparoscopic Cholecystectomy (LC) is reported to be higher as compared to Open Cholecystectomy. Studies have shown varying degree of success in reducing BDI by using Critical View of Safety (CVS) technique before clipping and cutting any structure. In this study, we will see whether CVS technique is faster and safer compared to conventional infundibular technique.

**Methods:**

This comparative study was conducted on patients who presented to Surgical Out-Patient-Department (OPD) of Khyber Teaching Hospital from July 2015 to June 2016. Total of 438 patients were divided into two groups. Group-A in which LC was done using infundibular while in Group-B, CVS technique was utilized. Two groups were compared for operating time and BDI.

**Results:**

The operative time was significantly reduced for LC using CVS technique (50 mins vs. 73 mins). Minor leaks were comparable (0.5% vs. 0.9%) but there was a significant difference in major LEAKS between the two techniques (0.5% vs. 1.4%).

**Conclusion:**

Although the “critical view of safety” requires more dissection as compared to infundibular technique, but once learnt and mastered, it is faster and safer identification technique during laparoscopic cholecystectomy.

## INTRODUCTION

Since its conception, Laparoscopic Cholecystectomy (LC) has gained worldwide popularity as the “Gold Standard” procedure for Cholecystectomy. However despite its rapid acceptance and international glory, a major drawback has been seen in the form of bile duct injuries (BDI). In the initial days of LC, the rate of BDI was reported in the range of 0.5 to 2.7% as compared to Open Cholecystectomy 0.1-2.5%.^1^-^4^ This high BDI rates were explained by saying that it reflects the “learning curve”. But despite increase in expertise in laparoscopy, rate of BDI has remained static.^5^ Couple of large studies which included about one million cholecystectomies showed BDI rate of about 0.23%.^6^,^7^

There are multiple factors contributing to BDI. One study stated that misinterpretation of anatomy was the major factor cited by 92.7% surgeons while lack of experience was mentioned by 70.9% surgeons.^8^ A 30 day morbidity rate after LC is 9.84% which requires definite improvement.^6^

The cystic duct and artery are the only structures that require division; hence the objective of dissection is to identify these structures conclusively. Several methods of identification are in use. Some surgeons uses traditional method of identification which is the “Infundibular technique” of anatomical display. Strassberg^9^ in early 1990s introduced Critical View of Safety (CVS) technique for identification and claimed that this identification technique will greatly reduce BDI rates.

After its introduction, this CVS method was adopted by many surgeons. Averginos et al. in 2009 published the results of 1046 cholecystectomies without BDI using the CVS method. Only five patients had transient biliary leaks in the postoperative period which subsided within two to 14 days.^10^ Similarly; Yegiyants and Collins analyzed 3,000 patients and reported one BDI only.^11^ Sanjay et al. in 2010 studied using CVS technique after no BDI in 447 cholecystectomies and reported no BDIs.^12^

The objective of this study was to compare the traditional “Infundibular Technique” with the “Critical View of Safety” method for minimizing BDI’s in LC at a local level in terms of operative time and BDI.

## METHODS

This was a comparative study conducted during July 2015 to June 2016 on patients who presented to Surgical Out-Patient-Department (OPD) of Khyber Teaching Hospital with symptomatic Gall Stones subsequently confirmed by clinical examination and ultrasonography. The inclusion criteria were patients with Gall Stones, American Society of Anesthesiologist’s class I and those willing to participate in the study after a written informed consent. Patients with previous history of abdominal surgery and those unfit for pneumoperitoneum due to cardiac or pulmonary causes were excluded.

During one year duration, total of 438 patients were selected after they consented to be included in the study and after applying exclusion criteria. Those qualified for the study were given admission number. Those given odd number were grouped into group A which were operated by a laparoscopic surgeon who routinely uses infundibular technique. Those given even number were grouped into group B which were operated by the laparoscopic surgeon who uses critical view of safety technique.

Patient in Group-A underwent LC following the Infundibular technique while Group-B patients underwent LC with critical view of safety (CVS) technique.

In “Infundibular technique”, surgeon would establish the continuity of cystic duct (CD) with the infundibulum of the Gall Bladder (GB). Once done, it would be considered with surety that it is CD and clipped and cut.

In critical view of safety, three criteria are required to achieve this; the first states that the hepatocystic triangle; defined as the triangle formed by CD, the CHD, and inferior edge of the liver is cleared of fat and fibrous tissue. The CBD and CHD do *not* have to be exposed. The second states that the lower 1/3rd of the GB is separated from the liver to expose cystic plate. The third states that two and only two structures should be seen entering the GB.^13^,^14^

The procedures were performed by two consultant surgeons with a case experience of more than 100 LC. One uses infundibular technique while the other uses CVS technique. All patients were operated under General anesthesia using three ports technique, a 4^th^ or 5^th^ port was inserted in cases with difficult dissection. All patients were given pre operative prophylactic antibiotics upon induction. The duration of surgery (from time of insertion of 1^st^ port to the time of retrieval of GB) and any intra-operative complications were noted.

Post operatively patients were assessed using clinical examination for signs and symptoms of biliary leakage such as abdominal distention, fever, jaundice while sometimes liver function test and Ultrasound abdomen were needed to establish the bile leak. In those cases where drain was placed revealed bile leak. (Above highlighted methods were complementary to each other in confirming the fact that there is bile leak). All patients received adequate analgesia and were discharged once stable. The patients were called for follow up after 10 days for removal of stitches and to look for any jaundice due to biliary strictures. BDI were divided based on McMahon et al. classification whereby lacerations under 25% of CBD diameter or cystic-CBD junction was classified as minor injury, where as transaction or laceration over 25% of CBD diameter and postoperative bile duct stricture were classified as major injury.^3^ This was assessed by MRCP (magnetic resonance cholangio-pancreatography) and ERCP (Endoscopic Retrograde Cholangio-pancreatography).

**Table-I:** Demographic characteristics.

	Group-A: Infundibular technique N= 220		Group-B= CVS technique N= 218	
Gender	Female = 150 (68%)	Male = 70 (31.8%)	Female= 145 (66.5%)	Male=73 (33.5%)
Age	Mean = 33±3.5		Mean= 34±2.8	

**Table-II:** Comparison of Infundibular (Group A) and CVS (Group B) technique

	Group-A	Group-B
Average time consumed	45-120 mins (mean 73±2.3)	30-75 mins (mean 50 ±1.5)
Minor leak <25% of CBD	02 patients (0.90%)	01 patient (0.5%)
Major leakage > 25% of CBD or obstructive jaundice needing ERCP	03 patients (1.4%)	01 patient (0.5%)

All data was entered and analyzed by SPSS version 20.0. Qualitative data like gender and biliary leakage were measured in frequencies and percentages and presented as n (%). While quantitative/ numerical variables like age and duration of surgery were presented as Mean ± SD. All data was calculated with 95% confidence interval.

## RESULTS

Out of 438 patients, 220 underwent LC using Infundibular technique and were labeled as Group-A while 218 underwent LC using Critical view of safety technique and was labeled as Group-B.

## DISCUSSION

Surgeons have long strived to make LC the safest and complication free procedure; much of this effort has been made fruitful by the introduction and application of CVS. As seen in our study; the operative time is significantly reduced for patients undergoing a LC with CVS technique (50 mins vs. 73 mins). Vettoretto et al. and Viswanathan V also found significant differences in operative times of both procedures (51.5 min vs. 69.7 min) and (55.7 min vs. 74 min) respectively, which is comparable to our study.^15^,^16^

Another important aspect as pointed out by Lam T and Manatakis DK in separate studies is that there is negligible difference in achieving adequate CVS scores with operator experience (consultant vs. trainees); without adding significant operative time in the hands of trainees, thus advocating this technique for teaching and being safe regardless of surgeons experience.^17^,^18^

As observed in our study, there was no mortality in both the techniques. Morbidity including minor leaks were comparable (0.5% vs. 0.9%) but there was a significant difference in major morbidity between the two techniques (0.5% vs. 1.4%). This finding is consistent with international studies where the rate of major BDI was 0.1% vs. 0.2%.^15^

Currently, the CVS technique is accepted as a Gold Standard for reducing morbidity and mortality associated with LC by the European Association of Endoscopic Surgery (EAES).^19^,^20^ There are no randomized controlled trials published up-to-date to give us level-1 evidence that CVS prevents bile duct injuries.^10^,^11^ However, if we look at the large case series^10^,^11^,^21^ published so far, we believe that by strictly adhering to all the three criteria of CVS, BDI may be prevented because it helps in giving reliable exposure and identifying important structures of calot’s triangle.

## CONCLUSION

Although the “critical view of safety” requires more dissection as compared to infundibular technique, but once learnt and mastered, it is faster and safer identification technique during laparoscopic cholecystectomy. The CVS approach to LC should be incorporated into national guidelines and made mandatory, especially for the training of surgical residents.

### Authors’ Contribution

**MZK, MAK and MAK** conceived, designed the study, and edited the manuscript.

**MZK and SAM** were the operating surgeons.

**MAK and MAK** did data collection, statistical analysis and manuscript writing.

**SAM** edited the manuscript and prepared the final version for approval.

**MAK** made changes to the manuscript which were jointly approved by all the authors.
